# Induction of ferroptosis by ATF3 elevation alleviates cisplatin resistance in gastric cancer by restraining Nrf2/Keap1/xCT signaling

**DOI:** 10.1186/s11658-021-00271-y

**Published:** 2021-06-07

**Authors:** Dazhi Fu, Chunxiao Wang, Lei Yu, Rui Yu

**Affiliations:** 1 Department of General Surgery, First Affiliated Hospital of China Medical University, Liaoning Province Shenyang City Heping District Nanjingbei Road 155, Shenyang, 110001 Liaoning People’s Republic of China; 2 Department of General Surgery, Liaoning Health Industry Group, Benxi Iron & Steel Group, General Hospital, Benxi, 117000 Liaoning People’s Republic of China

**Keywords:** Gastric cancer, ATF3, Cisplatin resistance, Ferroptosis, Nrf2/Keap1 signaling

## Abstract

**Background:**

Currently, resistance against cisplatin (DDP) is a frequent problem for the success of advanced gastric carcinoma (GC) chemotherapy. Here, we sought to investigate the function of activating transcription factor 3 (ATF3) n GC chemoresistance.

**Methods:**

Expression of ATF3 was determined in GC cell lines (MNK45, SGC7901, and BGC823) and cisplatin (DDP)-resistant cells (SGC7901/DDP and BGC823/DDP). Biological informatics was performed to analyze ATF3 expression and prognosis in GC patients. Cisplatin resistance was evaluated. Ferroptosis was detected after ATF3 transfection of cells. The underlying molecular mechanism was also investigated.

**Results:**

Transcripts of ATF3 were decreased in GC cells and GC tissues. Kaplan–Meier plotter analysis revealed that ATF3 expression was positively related to the overall survival of GC patients. In particular, lower levels of ATF3 were observed in cisplatin-resistant SGC7901/DDP and BGC823/DDP relative to their parental cells. Notably, ATF3 elevation sensitized cisplatin-resistant cells to cisplatin. Mechanically, compared with parental cells, SGC7901/DDP and BGC823/DDP cells exhibited lower ferroptosis evident by lower ROS, MDA and lipid peroxidation and higher intracellular GSH levels. However, ATF3 elevated ferroptosis in SGC7901/DDP and BGC823/DDP cells. Intriguingly, ATF3 overexpression together with ferroptosis activator erastin or RSL3 treatment further enhanced ferroptosis and cisplatin resistance; however, the ferroptosis suppressor liproxstatin-1 reversed the function of ATF3 in ferroptosis and cisplatin resistance. Additionally, cisplatin-resistant cells exhibited stronger activation of Nrf2/Keap1/xCT signaling relative to parental cells, which was restrained by ATF3 up-regulation. Importantly, restoring Nrf2 signaling overturned ATF3-mediated ferroptosis and cisplatin resistance.

**Conclusion:**

ATF3 may sensitize GC cells to cisplatin by induction of ferroptosis via blocking Nrf2/Keap1/xCT signaling, supporting a promising therapeutic approach for overcoming chemoresistance in GC.

## Background

Gastric cancer (GC) currently ranks as the fifth most prevalent malignancy and the third in cancer-related mortality in modern society [1, 2]. Globally, GC poses a considerable health threat because epidemiological investigation confirms over 1 million estimated new cases of GC and almost 800,000 deaths annually [3]. Over 70% of GC cases occur in developing countries including China [4]. Currently, traditional radical surgery and palliative chemotherapy remain the mainstay of GC treatment. Cisplatin (DDP)-based chemotherapy is deemed as a common first-line treatment for cancer patients including GC. Nevertheless, GC patients with advanced disease usually acquire resistance to chemotherapy, resulting in a somber prognosis with a median overall survival of 8–11 months [5].

Cell death is crucial for the prevention of hyperproliferative diseases including cancer. Increasing cancer cell sensitivity to chemotherapy usually originates from cancer cell death. Ferroptosis was coined in 2012 and was recently defined as a new form of cell death characterized by iron-dependent lipid peroxidation [6]. Interestingly, reactive oxygen species (ROS) and malondialdehyde (MDA) are fairly specific lipid peroxidation products and frequently defined as markers of ferroptosis. Ferroptosis can be activated in specific pathological states and its maladjustment has been implicated in several physiological and pathological processes, such as neurodegeneration, renal failure and lung injury [7, 8]. Of interest, cancer cells can adapt to an oxidative environment to control ferroptosis that may reshape the tumor niche to facilitate tumor growth and progression. For instance, inhibition of ferroptosis by activating nuclear factor (erythroid-derived)-like 2 (Nrf2) signaling facilitates brain tumor growth [9]. Importantly, emerging evidence has implicated ferroptosis in chemoresistance in several cancers [10–13]. In head and neck carcinoma, cystine deficiency induces ferroptosis evident by glutathione (GSH) depletion and lipid ROS accumulation and thereby overcomes cisplatin resistance [10]. Moreover, induction of ferroptosis by ginkgetin enhances the therapeutic efficacy of cisplatin in non-small-cell lung cancer [12]. Therefore, investigating the crosstalk between ferroptosis and chemoresistance has provided a new insight for tumor intervention.

Activation transcription factor 3 (ATF3) is a common member of the ATF/ cAMP response element-binding (CREB) protein family. As a stress-induced transcription factor, ATF3 is increased in obese mice, and targeting ATF3 exhibits suppressive efficacy towards lipid accumulation [14, 15]. Recently, ATF3 has become a subject of interest in carcinoma due to its frequent and aberrant expression in multiple cancers. However, according to existing investigations, the function of ATF3 is complicated and contradictory in carcinogenesis. For one thing, ATF3 can act as an oncogene to regulate the development of breast cancer and skin cancer [16, 17]. On the other hand, several studies substantiate the down-regulation of ATF3 in cancers and support it as a suppressor in oncogenesis [18, 19]. Additionally, ATF3 is responsible for chemoresistance in non-small cell lung carcinoma [20] and nasopharyngeal cancer [21]. In the current study, we sought to investigate the function of ATF3 in cisplatin resistance in GC cells. Notably, a recent study confirmed that ATF3 promotes ferroptosis in fibrosarcoma cells [22]. Therefore, we also investigated whether ferroptosis is involved in ATF3 function in GC chemoresistance and how ATF3 regulates ferroptosis during this process. Our findings suggested that ATF3 could sensitize GC cells to cisplatin by ferroptosis induction via blocking Nrf2/Keap1/xCT signaling. Thus, the present research may implicate ATF3 as a promising therapeutic candidate to overcome chemoresistance in GC.

## Materials and methods

### Cell lines and culture

Human gastric epithelial cells (GES-1) (No. BNCC353464) were obtained from the Beina Chuanglian Biotechnology Research Institute (Beijing, China). Human GC cell lines (MNK45, SGC7901 and BGC823) were bought from the ATCC (Rockville, MD, USA). All cells were kept in RPMI 1640 medium (HyClone, South Logan, UT, USA) supplemented with 10% fetal calf serum (FCS), 1% penicillin and streptomycin. For the generation of cisplatin (DDP)-resistant SGC7901 and BGC823 cells, the parental GC cells were treated with gradually increased concentrations of cisplatin as in previous reports [23]. Briefly, the parental SGC7901 and BGC823 cells in the log-growth phase were incubated in culture medium containing cisplatin (0.1 µg/ml final concentration). Fourteen days later, the cisplatin dose was gradually increased to 0.2 µg/ml for further incubation for 2 weeks. Then, cisplatin concentration was gradually elevated to 0.4 µg/ml and 0.8 µg/ml according to similar procedures until cells could maintain stable growth and repeated passage. The final prepared cells were cisplatin-resistant cells and defined as SGC7901/DDP and BGC823/DDP, respectively. For culture, all cells were maintained at 37 °C in a humidified incubator with 5% CO_2_ and 95% O_2_.

### Cell treatment

The GC cell lines (SGC7901, BGC823, SGC7901/DDP, BGC823/DDP) were stimulated with the indicated doses of cisplatin. Furthermore, the SGC7901/DDP and BGC823/DDP cells were treated with ferroptosis agonist erastin (0.8 µM), RSL3 (0.1 µM) or antagonist liproxstatin-1 (80 nM, both from Selleck, Houston, Texas, USA) before and during cisplatin exposure [11].

### Construction of expression plasmids

To generate the expressed plasmids, total RNA from GC cells was extracted using the TRIzol reagent (Invitrogen, Carlsbad, CA, USA), followed by the synthesis of the first-strand cDNA with the SuperScript II First Strand Synthesis System (Invitrogen). Then, the full-length ATF3 and Nrf2 cDNA was PCR amplified. Then, the prepared cDNA was digested with the restriction enzymes and cloned into pcDNA3.1(+) plasmids to obtain the recombinant pcDNA-ATF3 and pcDNA-Nrf2 vectors. Subsequently, cisplatin-resistant GC cells were seeded in 24-well plates and transfected with pcDNA-ATF3 and pcDNA-Nrf2 plasmids using Lipofectamine 2000 (Invitrogen). During this process, cells transfected with empty vectors were defined as the negative control. Approximately 48 h later, western blotting was conducted to evaluate the transfected efficacy of plasmids.

### Human Protein Atlas and Kaplan–Meier plotter assay

The protein expression of ATF3 in normal gastric tissue and GC tissues was analyzed online using the Human Protein Atlas (https://www.proteinatlas.org/). The correlation between ATF3 and survival rate in GC patients was evaluated online using the Kaplan–Meier plotter (http://kmplot.com/analysis/index.php). The Kaplan–Meier plotter database is an online system to analyze mRNA Affymetrix Genechip and RNA-sequencing datasets for GC patients. All information was obtained from the online database and not collected by ourselves.

### Quantitative RT-PCR (qRT-PCR)

After total RNA extraction and cDNA synthesis, real-time PCR was conducted using the SYBR Premix Ex Taq II Kit (TaKaRa, Dalian, China) to quantify the transcription of ATF3. The specific oligonucleotide primer sequences for ATF3 and GAPDH are shown in Table 1 and were bought from Shanghai Sangon Co., Ltd (Shanghai, China). The cycling parameters were as follows: 95 °C 10 min for initial denaturation; 94 °C or 15 s, 58 °C for 30 s, and 72 °C for 15 s for 40 cycles. All specimens were subjected to the ABI PRISM 7000 system (Applied Biosystems; Foster City, CA, USA) for PCR reaction. The expression of each gene was analyzed by the 2^−ΔΔCt^ equation. The quantification of target gene expression was conducted by introducing the internal control GAPDH.

**Table 1:** Primer sequences of qRT-PCR

Name	Primer sequences (5′–3′)	Product size (bp)
ATF3	Sense, 5′-CTGGAAAGTGTGAATGCTGAAC-3′ Anti-sense, 5′-ATTCTGAGCCCGGACAATAC-3′	117
GAPDH	Sense, 5′-CAAGAGCACAAGAGGAAGAGAG-3′ Anti-sense, 5′-CTACATGGCAACTGTGAGGAG-3′	102

### Cell viability evaluation

Parental GC cells and cisplatin-resistant SGC7901/DDP and BGC823/DDP cells treated with pcDNA-ATF3, pcDNA-Nrf2, erastin, RSL3 or liproxstatin-1 were incubated with cisplatin for 48 h. Then, 10 μl of Cell Counting Kit (CCK)-8 solution (Nanjing Jiancheng Bioengineering Institute, Nanjing, China) was supplemented into each well for 3 h. Afterwards, cell viability was assessed by capturing the absorbance at 450 nm using a spectrophotometer.

### Immunoblot assay

Cells were subjected to the indicated treatment and then lysed at 4 °C in a RIPA lysis buffer. After protein quantification by the BCA protein assay kit (Beyotime, Shanghai, China), 30 µg of protein was resolved by 12% SDS-PAGE and transferred electrophoretically to a PVDF membrane. Subsequently, membranes were hybridized with primary antibodies against human ATF3 (1:1000), Nrf2 (1:800), Keap1 (1:1000), and xCT (1:8000) (all from Abcam, Cambridge, UK, USA) overnight at 4 °C. After incubation with horseradish peroxidase-conjugated secondary antibody, the ECL reagent (Beyotime) was added to visualize the binding signals. Densitometric analysis of immunostained protein bands was conducted using a Gel Doc XR imaging system (Bio-Rad Laboratories, Hercules, CA, USA) and Image J software (National Institutes of Health).

### Cell death assay

Cell death evaluation was performed with the previously described Annexin V-FITC/PI staining method [24]. Briefly, cells under various treatments were collected and resuspended in 500 μl of Annexin V Binding buffer (Beyotime). Then, cells were further incubated with 10 μl of Annexin V-FITC and 5 μl of PI in the dark. The positively stained cells were then counted using a FACSAria II flow cytometer (BD Biosciences, USA) with Cell Quest software.

### Reactive oxygen species (ROS) measurement

Cellular ROS generation was measured by adding 20 µM of cell-permeating probe 2ʹ,7ʹ-dichlorodihydrofluorescein diacetate (DCFH-DA; Sigma). After incubation for 0.5 h, the produced fluorescence intensity was determined using a spectrofluorimeter at 485 nm excitation wavelength and 530 nm emission wavelength. All protocols were performed following the product instructions.

### Malondialdehyde (MDA) and glutathione (GSH) assay

The contents of MDA in cells were determined using a commercial MDA Detection Kit (Nanjing Jiancheng Bioengineering Institute). Briefly, cells that received various treatments were lysed to prepare supernatants. After that, the supernatants were reacted with 200 μl of MDA reaction solution at 100℃ for 15 min. Then, MDA levels were measured at 532 nm using a microplate reader. For cellular GSH detection, a commercial GSH Detection Kit (Nanjing Jiancheng Bioengineering Institute) was introduced. The absorbance at 420 nm was captured to calculate cellular GSH contents. All procedures were conducted according to instructions provided by manufacturers.

### Evaluation of lipid peroxidation

For intracellular lipid peroxidation assay, cells were collected and resuspended in PBS buffer. Then, cells were stained with 2.5 µM BODIPY-C11 fluorescent dye (Invitrogen) for 10 min in a cell culture incubator. After rinsing with PBS three times, all specimens were analyzed using a flow cytometer (BD Biosciences).

### Statistical analysis

Statistical calculations were carried out using SPSS 22.0 software. Data are shown as mean ± standard deviation (SD). The data shown in the figure represented at least three independent experiments. The significance of differences was analyzed using Student’s *t*-test for two experimental groups and ANOVA with the Student–Newman–Keuls test for three or more groups. A *p*-value < 0.05 was defined as statistically significant (*P < 0.05, **P < 0.01).

## Results

### Expression and prognosis of ATF3 in gastric cancer cells and tissues

**Fig. 1:**
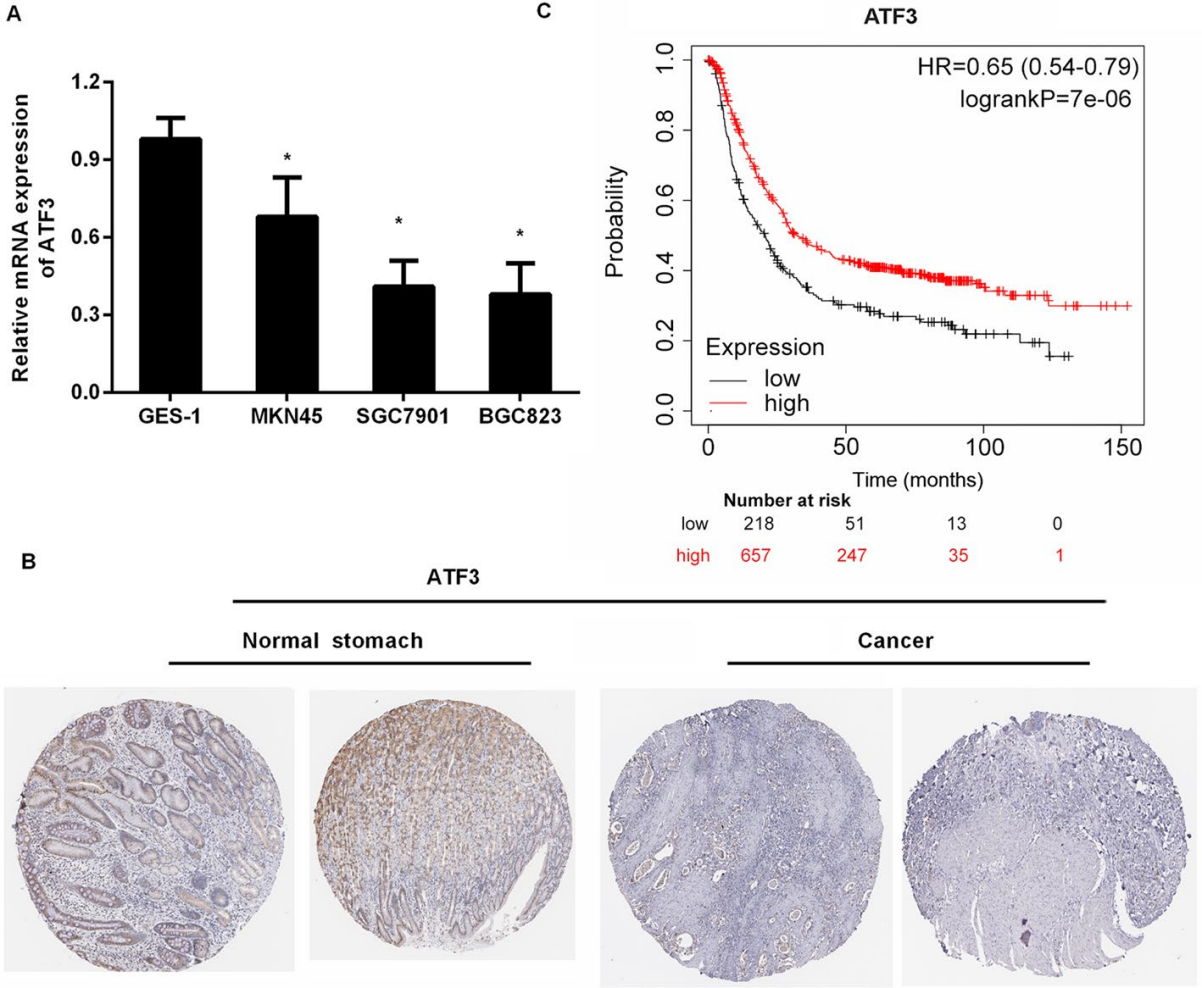
ATF3 expression and prognosis in gastric cancer cells and tissues. **A** Transcription of ATF3 in human gastric epithelial cell GES-1 and GC cell lines (MNK45, SGC7901 and BGC823). **B** ATF3 protein expression in normal stomach and GC tissues. Images were obtained from the Human Protein Atlas online database. **C** Kaplan–Meier plotter analysis evaluated ATF3 expression and overall survival in GC patients. *P < 0.05

To elucidate the function of ATF3 in GC, we first determined the expression of ATF3 in GC cells. As shown in Fig. 1A, in contrast to the human gastric epithelial cell GES-1, the mRNA levels of ATF3 were decreased in GC cell lines (MNK45, SGC7901 and BGC823). Lower transcription of ATF3 was observed in SGC7901 and BGC823 cells. Moreover, histochemistry data for GC using the Human Protein Atlas revealed that ATF3 protein was moderately expressed in normal gastric tissues, but weakly expressed in GC tissues (Fig. 1B). In Kaplan–Meier plotter analysis, GC patients with lower ATF3 expression had worse overall survival than those with higher ATF3 expression (Fig. 1C). These data indicate the critical roles of ATF3 in the progression of GC.

### Down-regulation of ATF3 is validated in cisplatin-resistant gastric cancer cells

**Fig. 2:**
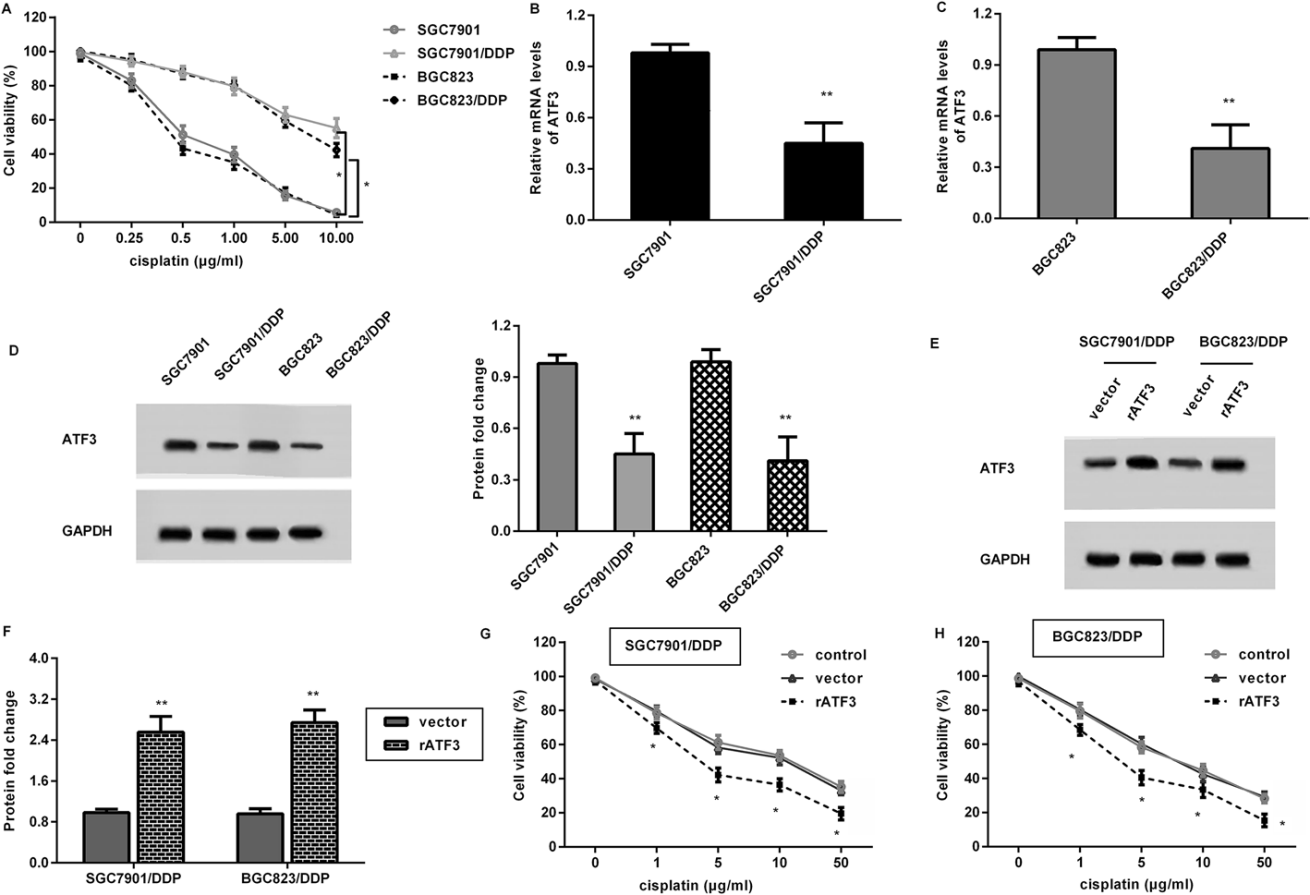
Overexpression of ATF3 sensitized gastric cancer cells to cisplatin. **A** Cisplatin-resistant cells (SGC7901/DDP and BGC823/DDP) and their parental cells (SGC7901 and BGC823 cells) were exposed to various doses of cisplatin. Then, a CCK-8 assay was performed to determine cell viability. **B**, **C** mRNA levels of ATF3 in cisplatin-resistant and parental GC cells. **D** Western blotting was conducted to analyze protein expression of ATF3. **E**, **F** SGC7901/DDP and BGC823/DDP cells were transfected with recombinant ATF3 plasmids, and the effect on ATF3 protein expression was then evaluated by western blotting. **G**, **H** After transfection with rATF3 vectors, cell viability under cisplatin exposure was then detected. *P < 0.05. **P < 0.01

As presented in Fig. 2A, cisplatin-resistant GC cells (SGC7901/DDP and BGC823/DDP) exhibited stronger resistance to cisplatin relative to the corresponding parental GC cells. Notably, compared with SGC7901 cells, lower transcription of ATF3 was observed in SGC7901/DDP cells (Fig. 2B). Moreover, an analogously lower ATF3 mRNA level was observed in BGC823/DDP cells relative to BGC823 cells (Fig. 2C). Similarly, the protein expression of ATF3 was decreased in SGC7901/DDP and BGC823/DDP, compared to their parental GC cells (Fig. 2D).

### ATF3 enhancement blunts gastric cancer cell resistance to cisplatin

To further investigate the potential roles of ATF3 in cisplatin resistance in GC, SGC7901/DDP and BGC823/DDP were transfected with recombinant ATF3 plasmids (rATF3) to overexpress ATF3 (Fig. 2E and F). Intriguingly, up-regulation of ATF3 dramatically blunted SGC7901/DDP (Fig. 2G) and BGC823/DDP cell (Fig. 2H) resistance to cisplatin. These results reveal that ATF3 elevation may blunt GC cell resistance to cisplatin.

### Elevation of ATF3 affects ferroptosis in cisplatin-resistant gastric cancer cells

**Fig. 3:**
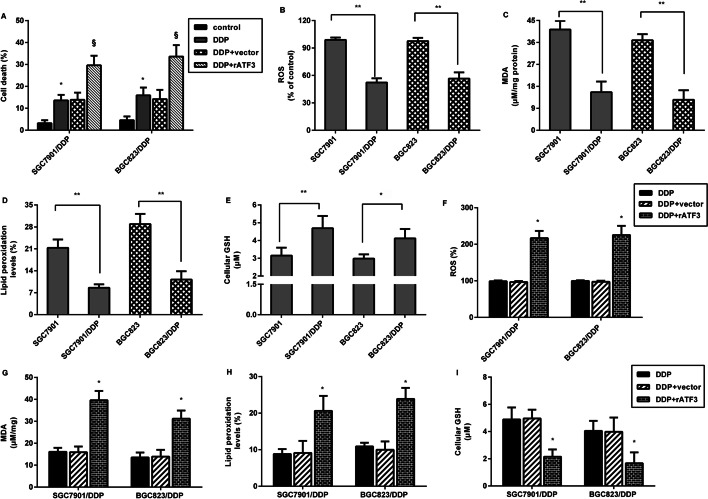
ATF3 evoked ferroptosis in GC cells. **A** SGC7901/DDP and BGC823/DDP cells transfected with ATF3 plasmids were exposed to cisplatin. Cell death was then assessed by Annexin V-FITC/PI. **B**–**E** Ferroptosis was evaluated in cisplatin-resistant and parental GC cells by detecting ROS (**B**), MDA (**C**), lipid peroxidation (**D**) and GSH levels (**E**). **F**–**I** Cisplatin-resistant GC cells were treated with ATF3 vector transfection and cisplatin exposure, and the ROS (**F**), MDA (**G**), lipid peroxidation (**H**) and GSH levels (**I**) were then measured. *P < 0.05. **P < 0.01

Emerging evidence corroborates the critical function of ferroptosis in tumor chemotherapy resistance including GC [10, 25, 26]. Therefore, we further investigated the correlation between ATF3 and ferroptosis in GC cells. As shown in Fig. 3A, ATF3 overexpression further aggravated cisplatin-evoked cell death in SGC7901/DDP and BGC823/DDP. Additionally, lower ROS (Fig. 3B), MDA (Fig. 3C), and lipid peroxide (Fig. 3D) levels were detected in parental SGC7901 and BGC823 cells relative to SGC7901/DDP and BGC823/DDP cells, concomitant with higher GSH levels (Fig. 3E). These data imply that cisplatin-resistant GC cells exhibited lower ferroptosis. Of interest, overexpression of ATF3 enhanced ROS (Fig. 3F), MDA (Fig. 3G) and lipid peroxidation (Fig. 3H) levels in SGC7901/DDP and BGC823/DDP cells. However, ATF3 enhancement inhibited GSH levels in SGC7901/DDP and BGC823/DDP cells. These findings suggest that ATF3 up-regulation may evoke ferroptosis in cisplatin-resistant GC cells.

### Ferroptosis accounts for the effects of ATF3 up-regulation against cisplatin resistance

**Fig. 4:**
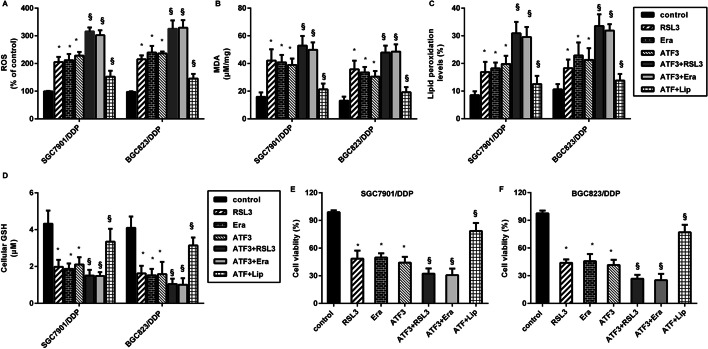
Ferroptosis was responsible for the effects of ATF3 up-regulation on cisplatin resistance. Cisplatin-resistant GC cells (SGC7901/DDP and BGC823/DDP) were treated with ATF3 transfection, ferroptosis agonist erastin, RSL3 and antagonist liproxstatin-1 under cisplatin conditions. Then, the levels of ROS (**A**), MDA (**B**), lipid peroxidation (**C**) and GSH (**D**) were determined. **E**, **F** Cell viability in SGC7901/DDP (**E**) and BGC823/DD (**F**) was evaluated by CCK-8 assay. *P < 0.05 vs. control group. ^§^*P* < 0.05 vs. ATF3-treated group

To further elucidate the involvement of ferroptosis in ATF3-mediated cisplatin resistance in GC cells, the ferroptosis agonist erastin, RSL3 and the antagonist liproxstatin-1 were applied. As presented in Fig. 4A–C, activators of erastin and RSL3 (inhibition of GPx4 to induce ferroptosis) markedly enhanced ROS, MDA production and lipid peroxidation. Intriguingly, ATF3 overexpression also enhanced ROS production in SGC7901/DDP and BGC823/DDP cells under cisplatin exposure; moreover, activation of ferroptosis by ATF3 overexpression together with the ferroptosis agonist erastin or RSL3 further elevated ROS levels. Nevertheless, the ferroptosis inhibitor liproxstatin-1 reversed ATF3-induced ROS production (Fig. 4A). Concomitantly, induction of ferroptosis further aggravated the effects of ATF3 on MDA (Fig. 4B), lipid peroxidation (Fig. 4C) and GSH levels (Fig. 4D); whilst suppression of ferroptosis by liproxstatin-1 overturned the above efficacy. Importantly, ATF3 elevation mitigated cell viability in cisplatin-treated SGC7901/DDP (Fig. 4E) and BGC823/DDP cells (Fig. 4F), which was further reversed by liproxstatin-1. Moreover, ATF3 up-regulation further aggravated ferroptosis activator erastin- or RSL3-inhibited cell viability. These data indicate that ATF3 sensitizes GC cells to cisplatin via induction of ferroptosis.

### Activation of Nrf2/Keap1/xCT signaling in SGC7901/DDP is restrained by ATF3 overexpression

**Fig. 5:**
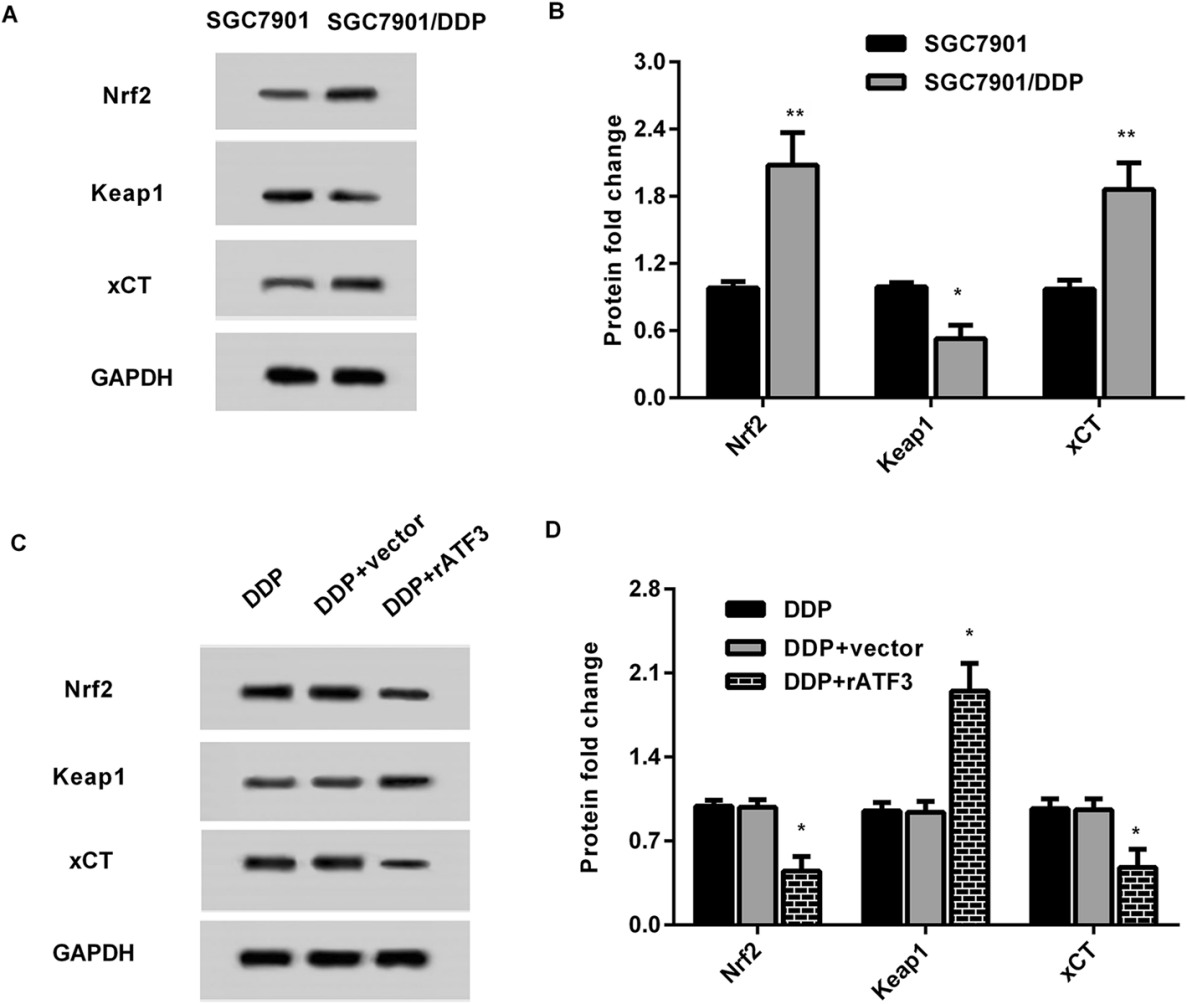
Up-regulation of ATF3 restrained the activation of Nrf2/Keap1/xCT signaling in SGC7901/DDP cells. **A**, **B** Protein expression of Nrf2, Keap1 and xCT was analyzed in SGC7901 and SGC7901/DDP cells. Image J software was used to quantify the density of the bands. **C**, **D** After transfection with ATF3 plasmids, activation of Nrf2/Keap1/xCT signaling was detected in SGC7901/DDP cells under cisplatin conditions. *P < 0.05. **P < 0.01

Recent studies have established that Nrf2/Keap1 signaling has a key role in the development of cancers including GC [9, 27]. Intriguingly, higher protein expression of Nrf2 and xCT was observed in SGC7901/DDP cells relative to SGC7901 cells, concomitant with the decrease in Keap1 protein levels (Fig. 5A and B). Moreover, compared with DDP groups, overexpression of ATF3 restrained this signaling by decreasing Nrf2 and xCT expression and increasing Keap1 protein expression (Fig. 5C and D).

### Nrf2/Keap1/xCT signaling is involved in ATF3-mediated ferroptosis and cisplatin resistance in gastric carcinoma

**Fig. 6:**
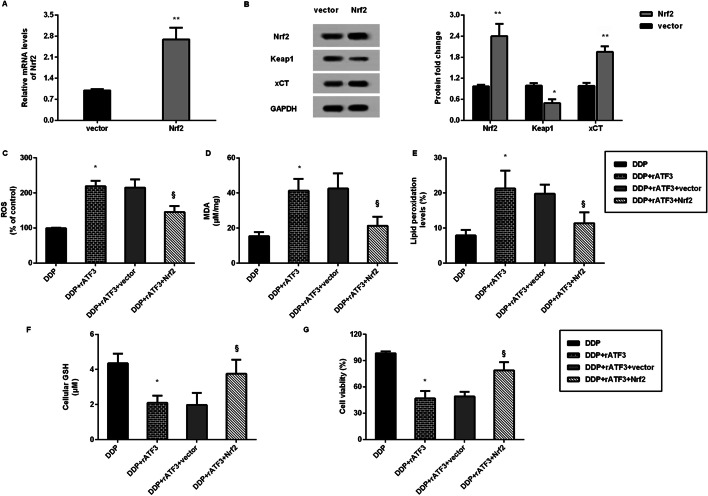
Nrf2/Keap1/xCT signaling involved in ATF3-mediated ferroptosis and cisplatin resistance in GC. **A** The effect of Nrf2 plasmid transfection on mRNA levels of Nrf2 was analyzed by qRT-PCR. **B** After transfection with Nrf2 vector, protein expression of Nrf2, Keap1 and xCT was measured. *P < 0.05, **P < 0.01 vs. vector group. **C** SGC7901/DDP cells were treated with ATF3 plasmids and/or Nrf2 plasmids before cisplatin exposure. Then, the levels of ROS (**C**), MDA (**D**), lipid peroxidation (**E**), GSH (**F**) and cell viability (**G**) were analyzed. *P < 0.05 vs. DDP group. ^§^*P* < 0.05 vs. DDP and ATF3 group

As shown in Fig. 6A, transfection with Nrf2 plasmids enhanced the transcription of Nr2 in SGC7901/DDP cells. Simultaneously, Nrf2 vector transfection increased protein expression of Nrf2, xCT and inhibited the Keap1 protein levels, indicating the restoration of Nrf2 signaling (Fig. 6B). Notably, ATF3 elevation enhanced ROS production (Fig. 6C), MDA levels (Fig. 6D) and lipid peroxidation (Fig. 6E), and decreased GSH levels (Fig. 6F). Nevertheless, reactivating Nrf2 signaling reversed the ATF3-mediated above effects (Fig. 6C–F), indicating that ATF3 may trigger ferroptosis by blocking the Nrf2/Keap1 pathway. Additionally, restoring the Nrf2 pathway increased cell viability in ATF3-overexpressed SGC7901/DDP cells (Fig. 6G).

## Discussion

Gastric cancer (GC) represents a global health threat due to cancer-evoked death, and a majority of people with GC are usually diagnosed at an advanced stage. ATF3 has moved into the limelight within cancer research, where its expression is dysregulated in multiple cancer types; however, its expression and function are contradictory. Several studies corroborate obvious down-regulation and suppressive efficacy of ATF3 in carcinogenesis, including hepatocellular carcinoma [19], prostate cancer [18], and non-small cell lung carcinoma [20]. Conversely, ATF3 exhibits high expression levels in breast and skin carcinomas, and can act as an oncogene to participate in carcinogenesis [15, 16]. In this study, we confirmed the decrease of ATF3 in GC cells relative to GES-1 human gastric epithelial cells. Importantly, histochemical data from the Human Protein Atlas revealed weak expression of ATF3 in GC tissues. Furthermore, Kaplan–Meier plotter analysis corroborated that ATF3 expression was positively related to the overall survival of GC patients. Therefore, these findings indicate the potential suppressive roles of ATF3 in GC.

Palliative chemotherapy is the mainstay for most patients with GC; nevertheless, poor or even no response to chemotherapy is often observed in the clinical treatment of GC patients due to acquired drug resistance that frequently results in a low 5-year survival rate [5]. In this study, we constructed cisplatin-resistant GC cells and substantiated the decrease of ATF3 in cisplatin-resistant cells relative to their parental cells, implying the potential of ATF3 in cisplatin resistance in GC. Importantly, ATF3 elevation sensitized cisplatin-resistant GC cells to cisplatin. Analogously with this finding, former research indicated that ATF3 accounts for the reinforcing effects on cisplatin cytotoxicity in non-small cell lung carcinoma [20]. Similarly, ATF3 promotes nasopharyngeal cancer cell sensitivity to paclitaxel [21]. Additionally, overexpression of ATF3 also enhances radioresistance of breast cancer [28].

We next corroborated one noteworthy finding that ATF3 elevation enhanced cell death in cisplatin-resistant GC cells. Concomitantly, cisplatin-resistant GC cells exhibited lower ROS, MDA, lipid peroxidation and higher GSH levels relative to parental cells, indicating the lower ferroptosis in cisplatin-resistant GC cells. Ferroptosis was recently defined as a newly discovered form of cell death that is a nexus between redox biology, metabolism, iron-dependent lipid peroxidation and health [29]. Unlike apoptosis, ferroptosis does not involve the activation of caspase. Ferroptosis can evoke ROS and MDA increase, and ultimately results in overwhelming lipid peroxidation to cause cell death [7]. Currently, fast-growing studies of ferroptosis in cancer have supported a novel viewpoint that induction of ferroptosis may ultimately aid in the discovery of novel therapeutic strategies to improve cancer treatment [25, 30]. For instance, ferroptosis triggered by tanshinone IIA or ferroptosis agonist erastin evokes GC cell death, indicating a new insight for GC intervention [30]. Recent research confirms that cisplatin acts as an inducer for ferroptosis, which opens up a new way for their utility in clinical practice [13]. Currently, triggering ferroptosis is recognized as a promising strategy to eradicate aggressive malignancies that are resistant to traditional chemotherapy [25]. Corroborating previous research [11, 24], we found that cisplatin-resistant GC cells exhibited lower ferroptosis. In this study, ATF3 elevation induced ferroptosis in cisplatin-resistant GC cells. Notably, a recent study confirmed the pro-ferroptosis efficacy of ATF3 in fibrosarcoma cells [22]. Importantly, ATF3 elevation evoked ferroptosis and ultimately sensitized cisplatin-resistant cells to cisplatin, whilst the antagonist liproxstatin-1 overturned ATF3 effects on ferroptosis and subsequent cisplatin resistance. These findings highlight that ATF3 may blunt cisplatin-resistant GC cells to cisplatin by evoking ferroptosis. Analogously, ferroptosis suppression promotes chemoresistance in GC [26].

Mechanistically, the activation of Nrf2/Keap1/xCT signaling was observed in cisplatin-resistant GC cells; however, ATF3 elevation restrained this activation. Nrf2 is a cytosolic transcription factor that can bind and suppress Keap1 expression to regulate antioxidant and stress-related events. In normal conditions, activation of the Nrf2/Keap1 pathway induces cytoprotection and antagonizes tissue injury [31, 32]. Over the past few years, abundant research has substantiated tumor suppressor efficacy of Nrf2 in carcinogenesis [27]. Contradictorily, inhibition of Nrf2 activation has recently been considered as a promising approach to suppress tumor growth and overcome chemoresistance [27]. Intriguingly, recent research has implicated Nrf2 signaling in restraining lipid peroxidation and ferroptosis [9, 33]. In the current study, reactivating Nrf2/Keap1/xCT signaling overturned ATF3-mediated suppression in ferroptosis and subsequent cisplatin resistance in resistant GC cells. Suppression of xCT usually leads to GSH depletion and finally results in ferroptosis in cancer cells [22, 25]. Notably, activation of the Nrf2/Keap1 pathway increases xCT (SLC7A11) expression and diminishes ferroptosis, thus facilitating glioma cell growth [9]. Especially, blockage of the Nrf2 pathway overcomes resistance of cisplatin-resistant head and neck cancer cells to cisplatin by inducing ferroptosis[24]. Thus, these observations prompt us to conclude that Nrf2/Keap1/xCT-mediated ferroptosis may account for the efficacy of ATF3 against cisplatin resistance in GC cells. As expected, restoring the Nrf2 signaling muted ATF3-mediated ferroptosis and cisplatin resistance in GC cells. Notably, in this study, we confirmed the correlation between ATF3 and NRf2 in ferroptosis inhibition in GC. However, what is the potential mechanisms by which ATF3 blocks NRF2? A recent study confirmed that ATF3-mediated suppression is a consequence of direct ATF3-Nrf2 protein–protein interactions [34]. Therefore, does ATF3 directly affect Nrf2 expression by protein–protein interaction, or indirectly regulate Nrf2 expression by other common pathways? All of these questions are still undefined and should be explored in our future research.

## Conclusions

Collectively, one noteworthy observation in the current study was that expression of ATF3 was decreased in GC cells and tissues, and was positively related to the overall survival of GC patients. Notably, lower expression was observed in cisplatin-resistant cells relative to their parental cells. Especially, the current study highlighted a novel finding that elevation of ATF3 sensitized cisplatin-resistant GC cells to cisplatin by evoking ferroptosis via inhibition of Nrf2/Keap1/xCT signaling. Consequently, the present data may support a promising therapeutic approach to overcome cisplatin resistance in the treatment of GC patients.

## Data Availability

All data generated or analyzed during this study are included in this published article.

## Abbreviations

DDP: Cisplatin
GC: Gastric carcinoma
ATF3: Activating transcription factor 3
ROS: Reactive oxygen species
MDA: Malondialdehyde
I/R: Ischemia–reperfusion
Nrf2: Nuclear factor (erythroid-derived)-like 2
GSH: Glutathione
